# Streamlined miRNA loading of surface protein-specific extracellular vesicle subpopulations through electroporation

**DOI:** 10.1186/s12938-024-01311-2

**Published:** 2024-11-21

**Authors:** Corinna Torabi, Sung-Eun Choi, Thomas R. Pisanic, Michael Paulaitis, Soojung Claire Hur

**Affiliations:** 1 Department of Mechanical Engineering, Johns Hopkins University, 3400 N Charles Street, Baltimore, MD 21218 USA; 2 RASyn, LLC, 700 Main Street, Cambridge, MA 02139 USA; 3 Institute for NanoBioTechnology, Johns Hopkins University, 3400 N Charles Street, Baltimore, MD 21218 USA; 4 Department of Oncology, Johns Hopkins University, 600 N Wolfe St, Baltimore, MD 21287 USA; 5 Center for Nanomedicine at Wilmer Eye Institute, Johns Hopkins University School of Medicine, Baltimore, MD USA; 6 Sidney Kimmel Comprehensive Cancer Center, Johns Hopkins University, 401 N Broadway, Baltimore, MD 21231 USA

**Keywords:** Extracellular vesicles, Electroporation, Engineered extracellular vesicles, miRNA loading, Immunopurification, Targeted gene delivery, miRNA therapeutics

## Abstract

**Background:**

Extracellular vesicles (EVs) have emerged as an exciting tool for targeted delivery of therapeutics for a wide range of diseases. As nano-scale membrane-bound particles derived from living cells, EVs possess inherent capabilities as carriers of biomolecules. However, the translation of EVs into viable therapeutic delivery vehicles is challenged by lengthy and inefficient processes for cargo loading and pre- and post-loading purification of EVs, resulting in limited quantity and consistency of engineered EVs.

**Results:**

In this work, we develop a fast and streamlined method to load surface protein-specific subpopulations of EVs with miRNA by electroporating EVs, while they are bound to antibody-coated beads. We demonstrate the selection of CD81^+^ EV subpopulation using magnetic microbeads, facilitating rapid EV manipulations, loading, and subsequent purification processes. Our approach shortens the time per post-electroporation EV wash by 20-fold as compared to the gold standard EV washing method, ultracentrifugation, resulting in about 2.5-h less time required to remove unloaded miRNA. In addition, we addressed the challenge of nonspecific binding of cargo molecules due to affinity-based EV selection, lowering the purity of engineered EVs, by implementing innovative strategies, including poly A carrier RNA-mediated blocking and dissociation of residual miRNA and EV-like miRNA aggregates following electroporation.

**Conclusions:**

Our streamlined method integrates magnetic bead-based selection with electroporation, enabling rapid and efficient loading of miRNA into CD81^+^ EVs. This approach not only achieves comparable miRNA loading efficiency to conventional bulk electroporation methods but also concentrates CD81^+^ EVs and allows for simple electroporation parameter adjustment, promising advancements in therapeutic RNA delivery systems with enhanced specificity and reduced toxicity.

**Supplementary Information:**

The online version contains supplementary material available at 10.1186/s12938-024-01311-2.

## Introduction

Extracellular vesicles (EVs) are membrane bound nanoparticles secreted by all cells that play an important role in intercellular transport of biomolecules, including proteins, nucleic acids, and lipids. The ability of EVs to carry nucleic acids while protecting contents from degradation, coupled with reduced toxic side effects and off-target cargo accumulation compared to synthetic nanoparticles, makes EVs a clinically attractive strategy for the delivery of therapeutics [1–3]. EVs have particular advantages over synthetic lipid nanoparticles (liposomes) for therapeutic applications due to their unique native surface proteins facilitating tissue targeting and immune evasion [4]. Recent studies suggest that certain EV surface proteins enhance delivery efficiencies through immune evasion, tissue targeting, or improved cellular uptake [5–7]. For example, CD47, an integrin-associated transmembrane protein, enables EVs to avoid phagocytosis, prolonging their circulation time to better reach target tissues [8–10]. Similarly, tumor-derived EVs expressing the transmembrane intercellular adhesion molecule, CD54, have been shown to increase therapeutic-loaded EV uptake and accumulation in cancer cells [11]. In addition, tumor-derived EVs are known to have cancer-cell homing capabilities, potentially dictated by EV integrins, opening the opportunity for improved tumor-targeting mediated by naturally occurring EV membrane proteins [5, 12].

EVs have been shown to successfully deliver a wide range molecules with therapeutic applications, including siRNA [8, 13, 14], miRNA [15, 16], and drugs [17, 18]. In particular, miRNAs, which play a key role in regulating protein expressions, have prompted interest in loading functional exogenous miRNA into EVs for therapeutic applications [2], such as tumor suppression [19], promotion of angiogenesis to address diabetic wound healing [20], myocardial ischemia [16], and brain ischemia [21], and regulate inflammatory activation [22]. Engineered EVs also show great promise for treating neurological diseases, such as Parkinson’s disease, due to their ability to penetrate the blood–brain barrier [23].

While EVs have clear advantages, there remains a need for the development of new methods to better identify and select EVs expressing surface proteins that can enhance targeting [4, 24]. Indeed, a critical hurdle to the design and implementation of EVs is their inherent heterogeneity in size, structure, and molecular signatures due to diverse biogenesis pathways and cell origins [25]. Consequently, unlocking the full potential of EVs for targeted delivery faces challenges in achieving consistent cargo loading and selecting EVs with specific surface proteins for precise tissue targeting. In fact, clinical applications of EVs have been constrained by limitations in understanding how EV surface proteins impact gene and drug delivery. Current studies mostly employ heterogeneous EV populations derived from specific cell types or biological samples, an approach that may lead to difficulties in reproducibility and preclude the ability to study the delivery efficiency of distinct EV subpopulations [4, 5].

In addition, label-free EV purification methods, such as ultrafiltration, precipitation, and ultracentrifugation, are commonly chosen for EV-mediated drug delivery systems. However, these approaches lack specificity and often suffer from drawbacks such as long processing time, low purity, and structural damage to EVs [3, 24, 26]. The awareness of the importance of selecting clinically relevant, protein-specific EV subpopulations has spurred the development of immunopurification to distinguish and characterize distinct EV subpopulations and identify associated biomarkers [27–30]. However, these efforts have not yet explored methods to engineer unique populations of EVs optimized for delivery and therapeutic efficacy. Thus, the integration of EV enrichment strategies with EV loading processes is highly attractive, especially considering the emerging benefits of surface-protein-specific EV subpopulations for targeted therapeutic delivery systems.

Despite considerable effort, current EV loading methods [31, 32], such as incubation, parent cell genetic modification, and electroporation still produce heterogeneous loaded EVs with low purity [33]. This presents challenges in scalability, repeatability, and quality control for clinical applications. Electroporation, a versatile method compatible with a wide range of biomolecules, including small RNA [13, 34], DNA [35], and drugs [36], offers fast exogenous molecular loading. However, it is often coupled with inefficient, time-consuming, post-electroporation purification processes, such as the current gold-standard ultracentrifugation, which requires at least 70 min for pelleting and washing of EV samples. Moreover, electroporation is generally limited to bulk electroporation of a heterogenous EV population.

The inherent heterogeneity of EVs and the co-isolation of non-EV nanoparticles, which may induce undesirable immunogenicity in therapeutic interventions [4, 25], highlight the critical need for specific and efficient processes to reproducibly load and purify EVs for particular applications. Our innovation addresses this gap by providing a streamlined method that integrates surface marker-specific EV enrichment with efficient cargo loading, thereby enhancing specificity and efficiency for therapeutic RNA delivery. The utilization of affinity-based immunocapture creates CD81^+^ EV-microbead complexes (EMCs), enabling simple EV manipulation throughout the selection of target EV subpopulations, miRNA loading, and subsequent EV purification. We demonstrate a 20-fold decrease in time per EV wash and a 2.5 hour reduction in the overall time required for post-electroporation washing as compared to ultracentrifugation-based washing of EV samples. By employing blocking agents that minimize non-specific binding of RNA, along with RNase treatment, we establish an effective strategy for purifying miRNA-loaded EVs and minimizing unloaded residual cargo miRNA carryover, while also overcoming miRNA contamination related to inefficient, affinity-based EV purification. In addition, we confirm the integrity of the EV-membrane, proteins and loaded miRNA that can be compromised by freeze-thaw cycle and storage. Our approach demonstrates the ability to achieve electroporation loading of miRNA into pre-selected CD81^+^ EVs, producing EVs with enhanced loading efficiency compared to conventional loading of unsorted EVs. This novel approach opens avenues for more efficient and versatile EV-based applications, particularly for RNA-therapeutic delivery and analyses.

## Results

**Fig. 1:**
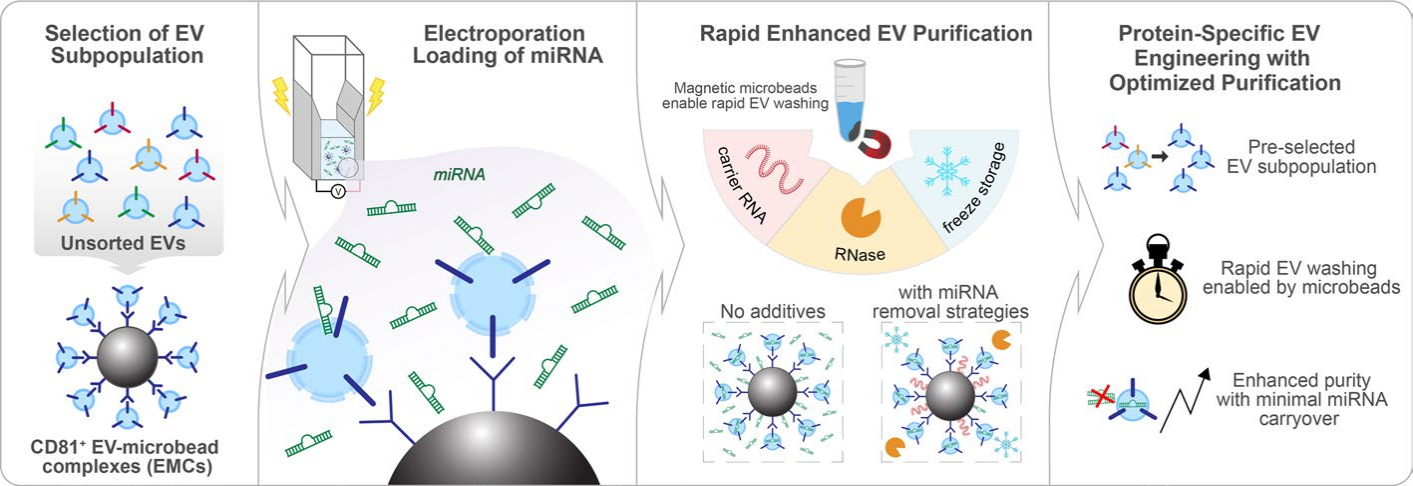
Schematic illustration of our streamlined workflow for production of miRNA-loaded extracellular vesicles. CD81^+^ EVs were selected from unsorted EVs derived from HEK-293 cells using anti-human CD81 antibody-coated microbeads to form CD81^+^ EV-microbead complexes (EMCs). CD81^+^ EMCs were subjected to electroporation for efficient loading of miRNA cargo into the EV subpopulation in a fast and integrated manner. To ensure high purity of loaded EVs from unloaded residual cargo miRNA, we implemented the use of poly A carrier RNA (cRNA) to reduce nonspecific binding of cargo miRNA onto affinity-binding sites of the EMCs. In addition, RNase washing was used to degrade unloaded cargo miRNA. Frozen storage of the EV subpopulations was likewise effective for further degrading unloaded cargo miRNA, enhancing the purity of miRNA-loaded CD81^+^ EVs

**Fig. 2:**
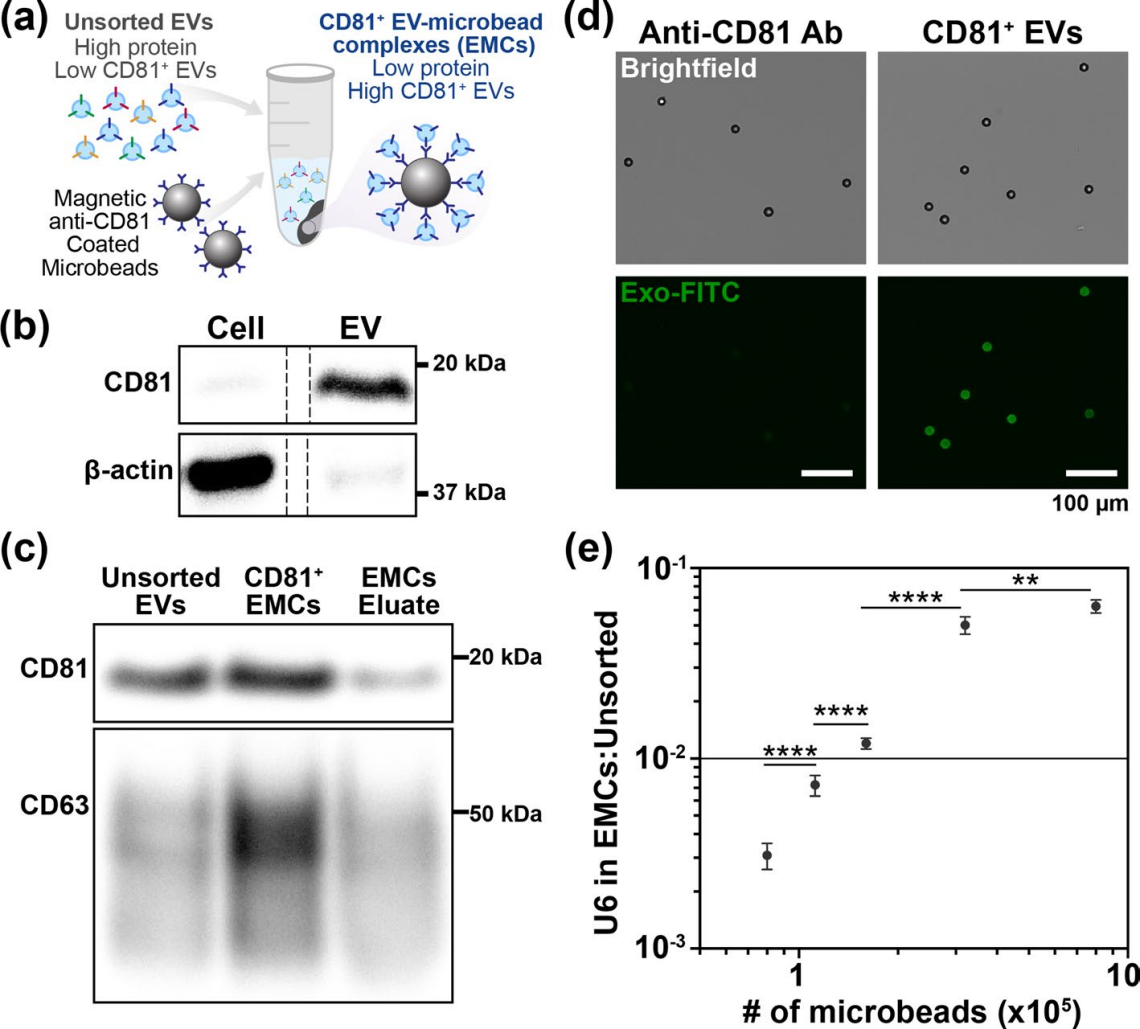
Characterization of EVs and CD81^+^ EV–microbead complexes (EMCs). **a** CD81^+^ EMCs are formed through antibody capture of CD81^+^ EVs from a heterogeneous EV population. **b** Protein enrichment in HEK-293 cell lysates and EVs isolated through polymer precipitation. 30 µg of protein were loaded per lane. The dotted line indicates that samples were run in non-adjacent lanes on the same gel. Full blot is shown in Figure S3. **c** Western blot analysis of CD81^+^ EMCs and the corresponding eluate containing unbound EVs. 25 µg of total protein containing unsorted EVs was used as a control. The CD81^+^ EMCs lane is loaded with protein from 6.4 × 10^5^ microbeads initially incubated with 200 µg of total protein, with the residual unbound protein being loaded into the EMC eluate lane. **d** Successful immunoprecipitation of CD81^+^ EVs on microbeads is visually validated by staining the EVs with a fluorescent dye. As a control, we used identical microbeads coated in anti-CD81 antibody and incubated with the fluorescent dye with no EVs. **e** U6 expression in CD81^+^ EMCs is normalized relative to U6 in 50 µg of total protein from isolated EVs and calculated using the 2^−∆Ct^ method. The Student’s *t* test (two-tailed) was performed to determine significance (**p ≤ 0.01, ****p ≤ 0.0001)

Our EV processing protocol involves microbead-based immunocapture of EVs that streamlines the selection, loading and purification of EVs (Fig. 1). Initially, a subpopulation of EVs expressing the surface protein CD81 was selectively captured on antibody-coated microbeads from a heterogeneous pool of unsorted EVs derived from HEK-293 cells, resulting in the formation of CD81^+^ EV-microbead complexes (EMCs), as shown in Fig. 2a. Electroporation-mediated loading of miRNA cargo, along with pre- and post-electroporation purification steps, was conducted with the EMCs remaining intact, facilitating easy manipulation of nanoscale, neutrally-buoyant EVs. This protein-specific EV-miRNA loading process harnesses the user-friendly attributes offered by antibody-coated magnetic microbeads and allows for preselection of EV subpopulations and rapid washing and manipulation, ultimately enhancing the final purity of EV samples.

### Purification and detection of unsorted EVs and CD81^+^ EV-microbead complexes (EMCs)

The presence and quantity of EVs harvested from HEK-293 cell culture media using precipitation isolation were assessed through total protein quantitation, immunoblotting of pan-EV surface proteins, and RT-qPCR amplification of an endogenous control gene. On average, total protein mass for EV isolates from 2.00 ± 0.22 × 10^7^ cells was 81.9 ± 3.12 µg of protein. Figure 2b shows protein expression levels of CD81 and β-actin present in an equivalent amount of total protein (30 µg/lane) from HEK-293 cells and EVs harvested from cell culture media. The distinct but inverse signal intensity of protein bands derived from EVs and their corresponding cellular origins, with high CD81 but faint β-actin signals from the controls containing and equivalent protein mass, suggests that the widely adopted EV-selection tetraspanin, CD81, can serve as an effective capture target for preselecting EVs from HEK-293 cells for subsequent electroporation [37]. Even with 30 µg of protein, the cellular housekeeping protein, β-actin, was nearly absent in the harvested EVs. This minimal β-actin signal, as well as the lack of consensus around a single internal reference protein to normalize EV proteins, compelled us to carefully maintain identical protein loading amounts when examining relative protein expressions for our experimental conditions [38].

Western blotting further confirmed the presence of CD81 in both CD81^+^ EMCs and unsorted EVs (Fig. 2c). The immunoprecipitation eluate (i.e., the unbound EVs) shows significantly lower CD81 expression compared to EMCs, indicating efficient enrichment of CD81^+^ EVs from the heterogeneous unsorted EV population. CD63, another commonly used tetraspanin for EV selection, was also highly enriched in the harvested EVs [39]. In addition, fluorescence microscopy successfully visualized CD81^+^ EVs, confirming successful extraction of the surface-protein-specific EVs using anti-CD81 antibody-coated microbeads (Fig. 2d). CD81^+^ EMCs exhibited a strong fluorescence signal when stained with a fluorescent EV dye, whereas no signal was observed in the anti-CD81-coated microbeads without EVs.

The immunocapture of CD81^+^ EVs from unsorted EV samples highlights that the protein mass of captured EVs represents only a fraction of the original unsorted EV population, which limits the common practice of using protein mass for normalizing EV input in electroporation reactions and emphasizes the need for alternative methods. To this end, we employed U6, a widely used endogenous control gene for normalizing miRNA in total EV populations during RT-PCR analysis [16, 21, 40, 41]. This approach ensures accurate assessment and comparison of CD81^+^ EMCs to unsorted EVs on the basis of small RNA content, since protein analyses do not directly correlate with PCR analyses. By implementing both Western blotting for protein analysis and U6 normalization for miRNA quantification, our approach ensured comprehensive and reliable assessment of EVs.

The use of the endogenous gene U6 allowed for a standard method of sample normalization for both unsorted EVs and CD81^+^ EMCs before and after miRNA loading. U6 expression levels were proportional to the number of EMCs, validating the effectiveness of U6 as an endogenous control for CD81^+^ EMCs (Fig. 2e). To maintain comparability with existing EV electroporation studies, we used 3 µg of unsorted EVs, aligning with the protein mass used in previous studies [10, 34, 35]. Subsequently, we determined that 1.6 × 10^5^ CD81^+^ EMCs carried a similar amount of EVs as 3 µg EV protein, as demonstrated by comparable U6 expression (3 µg unsorted EVs: C_T_ = 28.30 ± 0.10 and 1.6 × 10^5^ CD81^+^ EMCs: C_T_ = 28.79 ± 0.11).

### EMCs enable rapid EV manipulation and post-electroporation RNase incubation improves the removal of unloaded miRNA

**Fig. 3:**
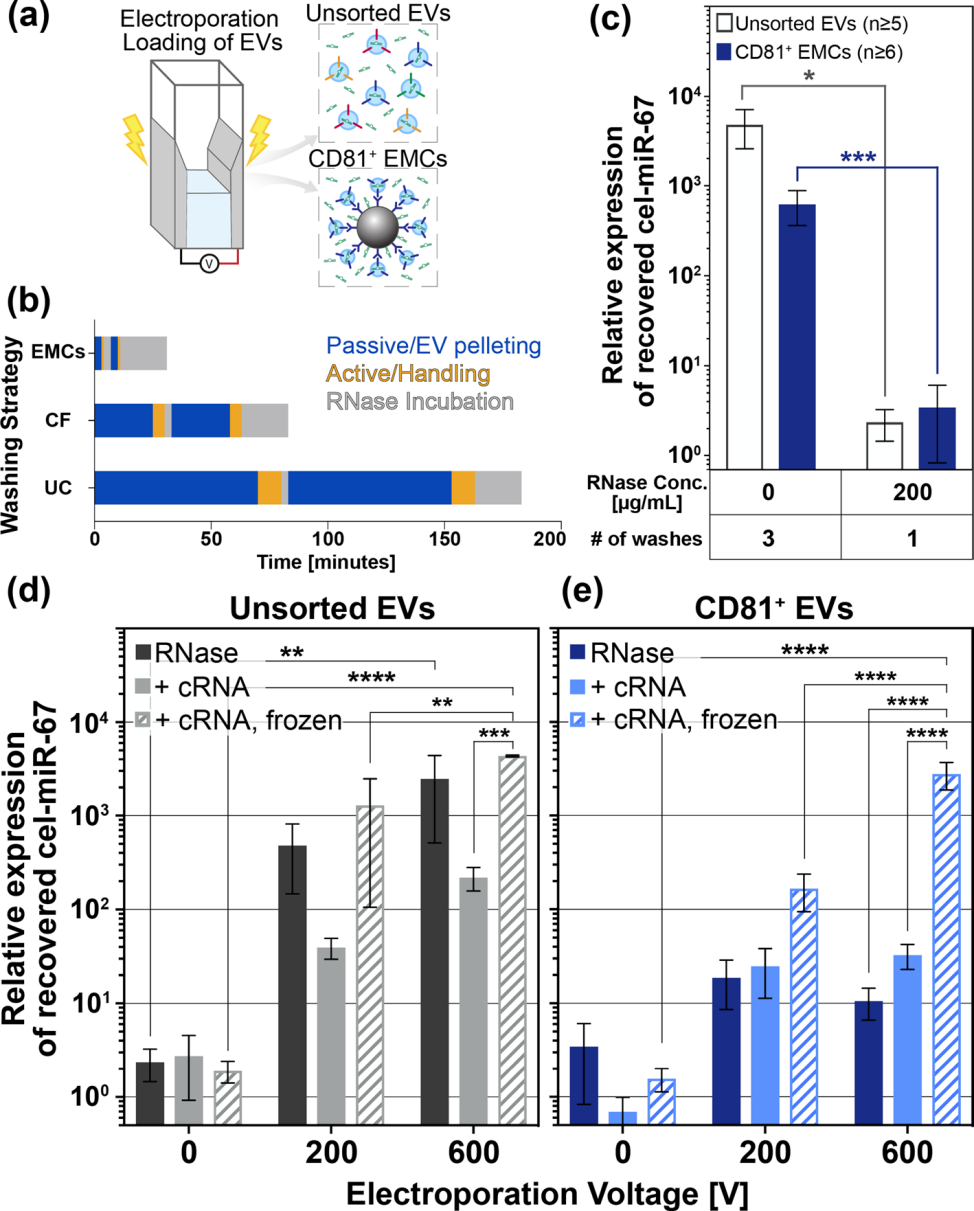
Effectiveness of strategies to remove unloaded residual cargo miRNA after electroporation loading. **a** Unsorted EVs and CD81^+^ EMCs are subjected to electroporation in a cuvette. Various purification methods were employed to remove unloaded cargo miRNA. **b** Overall post-electroporation processing time for removal of unloaded cargo miRNA from EMCs and unsorted EVs. Centrifugal filters (CF) and ultracentrifugation (UC) were used to wash unsorted EVs, which required longer pelleting time and active handling time. **c** The ratio of residual unloaded cargo miRNA (cel-miR-67) relative to the endogenous control gene (U6) was assessed under different EV washing conditions without electroporation: conventional PBS washing (0 µg/mL RNase) and washing with subsequent incubation with various RNase buffer concentrations. The Pfaffl method quantified relative expression, and Student’s *t* test (two-tailed) determined significance (*p ≤ 0.05 and ***p ≤ 0.001). Values are plotted as mean ± SEM. The relative expression of residual unloaded cargo miRNA detected from **d** unsorted EVs and **e** CD81^+^ EMCs, under applied voltages for electroporation (0 V, 200 V, and 600 V correspond to 0 kV/cm, 1 kV/cm, and 3 kV/cm, respectively). The cumulative effects on recovered miRNA with the addition of cRNA blocking and post-electroporation freezing for storage in addition to RNase washing only conditions are shown. The expression of cargo miRNA (cel-miR-67) relative to the endogenous control (U6) was calculated with the Pfaffl method using the 0 V condition as the control group. Values are plotted as mean ± SEM. Statistical significance is calculated with two-way ANOVA with Tukey’s multiple comparisons. Significance is shown as **p ≤ 0.01, ***p ≤ 0.001, and ****p ≤ 0.0001

Eliminating any residual unloaded cargo miRNA after electroporation loading is crucial to minimizing unintended miRNA carryover, reducing potential toxicity and ensuring accurate estimation of loaded cargo amounts, thus allowing for accurate assessment of therapeutic EV efficacy. This is particularly critical for therapeutic gene loading methods that utilize highly sensitive RT-qPCR gene amplification for quality control, which can be significantly skewed by minimal residual cargo molecules. Therefore, we systematically assessed miRNA carryover with respect to washing buffer formulation and the number of washes to identify optimal conditions for reducing residual miRNA for both unsorted EVs and CD81^+^ EMCs, as illustrated in Fig. 3a.

The conventional washing method involving sequential washes in PBS to remove unloaded miRNA was applied to both the unsorted EVs and CD81^+^ EMCs. Notably, the microbead carriers of CD81^+^ EVs facilitated rapid pelleting and manipulation, a process not applicable to unsorted EVs, which required lengthy ultracentrifugation runs to pellet the nanoscale EVs. CD81^+^ EMCs were pelleted in just 3 min, which is 20-fold faster per wash than the gold standard method of ultracentrifugation. This considerable time reduction per wash when using EMCs resulted in about 2.5 h less time required for the overall post-electroporation washing process as compared to using ultracentrifugation. Figure 3b and Table S1 show that the magnetic microbead carriers significantly reduced the time required for EVs pelleting and washing compared to the ultrafiltration and ultracentrifugation methods required for washing unsorted EVs. These substantial time savings markedly enhanced the processing efficiency of miRNA loading of EVs.

Despite the widespread use as a common EV washing method, repeated washing in PBS was insufficient for eliminating residual miRNA from both unsorted EVs and CD81^+^ EMCs. This limitation prompted us to explore RNase A to enzymatically digest unloaded RNA [15, 22, 42]. Washing and incubation of EVs in RNase A diluted in TE buffer (hereafter, RNase buffer) resulted in significantly lower levels of residual cargo miRNA compared to the conventional method of triple washes in PBS, demonstrating the effectiveness of washing in RNase buffer, as shown in Fig. 3c. Further optimization of RNase concentration and number of washes, shown in Figure S4a, revealed that an increase in the number of washes with 100 µg/mL RNase buffer from a single wash to triple washes followed by extended RNase incubation had no significant effect on the removal of unloaded miRNA. Similarly, increasing the RNase concentration from 50 to 500 µg/mL did not result in a significant reduction in the amount of unloaded miRNA (*p* > 0.05).

We also assessed the impact of RNase treatment on the internal control gene, U6, to ensure that the integrity of internalized RNA was maintained. We did not observe any degradation of U6 levels after single washes of RNase treatment, even at the highest RNase concentration of 500 µg/ml (Figure S4a,b), suggesting that RNA content internalized in EVs remained unharmed by the incubation in RNase buffer for 20 min at concentrations of ≤ 500 µg/mL. U6 levels for CD81^+^ EMCs washed three times were lower than for CD81^+^ EMCs washed only once (Figure S4c). These results suggest that the reduced U6 levels are a consequence of the loss of whole EVs during standard washing processes, rather than RNase degradation of RNA inside EVs.

Based on these results, we chose a single wash with RNase buffer to minimize sample loss and processing time, followed by a 20-min incubation in 200 µg/mL RNase buffer as the post-electroporation EV purification process. In fact, all tested RNase buffer concentrations were statistically indistinguishable. The similar results for all RNase concentrations were expected due to the marginal resistance of double-stranded RNA (cel-miR-67 miRNA mimic) to complete RNase degradation [43, 44], leading to slightly variable degradation of miRNA. Overall, washing both unsorted EVs and CD81^+^ EMCs one time in 200 µg/mL RNase buffer followed by an extended 20-min incubation period in RNase buffer significantly lowered the levels of unloaded residual miRNA.

### Presence of poly A carrier RNA minimizes nonspecific binding of cargo miRNA on antibody-coated microbeads

While providing unprecedently superior washing time, the use of antibody-coated beads as EV carriers for electroporation introduced a new challenge. Upon using RNase buffer to wash EVs after electroporation, we observed a lower electroporation efficiency in CD81^+^ EMC samples compared to unsorted EVs (Fig. 3d, e RNase). Further investigation revealed that affinity-based EV capture resulted in excessive nonspecific binding of miRNA to the microbead surface, leading to unintended carryover of unloaded miRNA. An investigation of antibody-coated microbeads, both alone and with EV conjugation, confirmed that microbeads coated only in antibodies enhanced nonspecific binding of miRNA as compared to the microbead surface coated in both EVs and antibody (Figure S5a). Washing with RNase buffer and a subsequent prolonged incubation in RNase buffer were not sufficient to completely remove nonspecifically-bound miRNA for CD81^+^ EMCs, complicating the ability to accurately evaluate our electroporation loading method.

To mitigate nonspecific binding of miRNA onto the antibody-coated microbead surface and false positive signals of cargo miRNA, we incorporated poly A carrier RNA (cRNA), rich in adenine nucleotides compared to the cargo miRNA (cel-miR-67), to passivate the microbead surfaces before the addition of cargo miRNA [45]. The reduction in nonspecific binding of cargo miRNA prevents unintended loss during electroporation as well as mitigating contamination by carryover of residual unloaded cargo miRNA post-electroporation. Importantly, the cRNA does not interfere with qPCR detection, suggesting that removal of the excess cRNA prior to adding cargo miRNA and performing electroporation was not necessary for miRNA loading. In addition, given its greater length than cargo miRNA, cRNA is expected to have much lower penetration potential through transient pores in the membrane of electroporated EVs compared to very short miRNA, decreasing the likelihood of cRNA competing with miRNA for entry into the EVs [35].

For CD81^+^ EMCs, a brief 5-min incubation with 25 µg/mL of cRNA proved to be effective in reducing nonspecific binding of miRNA to the microbead surface, as the cRNA selectively bound to exposed antibody-coated microbead surfaces, blocking sites where cargo miRNA could otherwise nonspecifically bind. As shown in Fig. 3e, the inclusion of cRNA diminished the presence of nonspecifically-bound miRNA at 0 V for CD81^+^ EMCs, emphasizing the successful electroporation at 200 V and 600 V that was previously obscured by the co-amplification of residual miRNA during qPCR quantification of internalized miRNA.

Conversely, in the case of unsorted EVs, the addition of cRNA appeared to reduce the efficiency of electroporation-mediated cargo miRNA loading compared to the condition where loaded-EVs were simply incubated in RNase buffer (Fig. 3d). This adverse impact of cRNA on the efficiency of miRNA loading in unsorted EVs is a consequence of a recognized phenomenon in which applied electric fields cause nucleic acids to form large, EV-sized, RNase-resistant aggregates, obscuring accurate assessment of EV electroporation efficiency [34]. Unlike CD81^+^ EMC samples, which benefit from rapid washes using magnetic beads, unsorted EVs rely on washing methods that inevitably co-pellet EV-sized RNA aggregates with the electroporated miRNA-loaded EVs. Consequently, while cRNA itself may not inherently inhibit qPCR amplification, the presence of remaining RNA aggregates may saturate the RNA-binding capacity during downstream RNA extraction processes, thereby reducing retrieval efficiency of miRNA internalized in EVs [46–48]. While this addition of cRNA may be unnecessary when engineering unsorted EVs through conventional methods, this finding demonstrates the importance of incorporating an effective purification step for loaded EV. In addition, these findings emphasize the added utility of microbead carriers in post-loading purification of engineered EVs to eliminate the co-collection of EV-sized, nucleic acid aggregates post-electroporation.

### Frozen storage of miRNA-loaded EVs further removes residual miRNA while maintaining EV membrane integrity

**Fig. 4:**
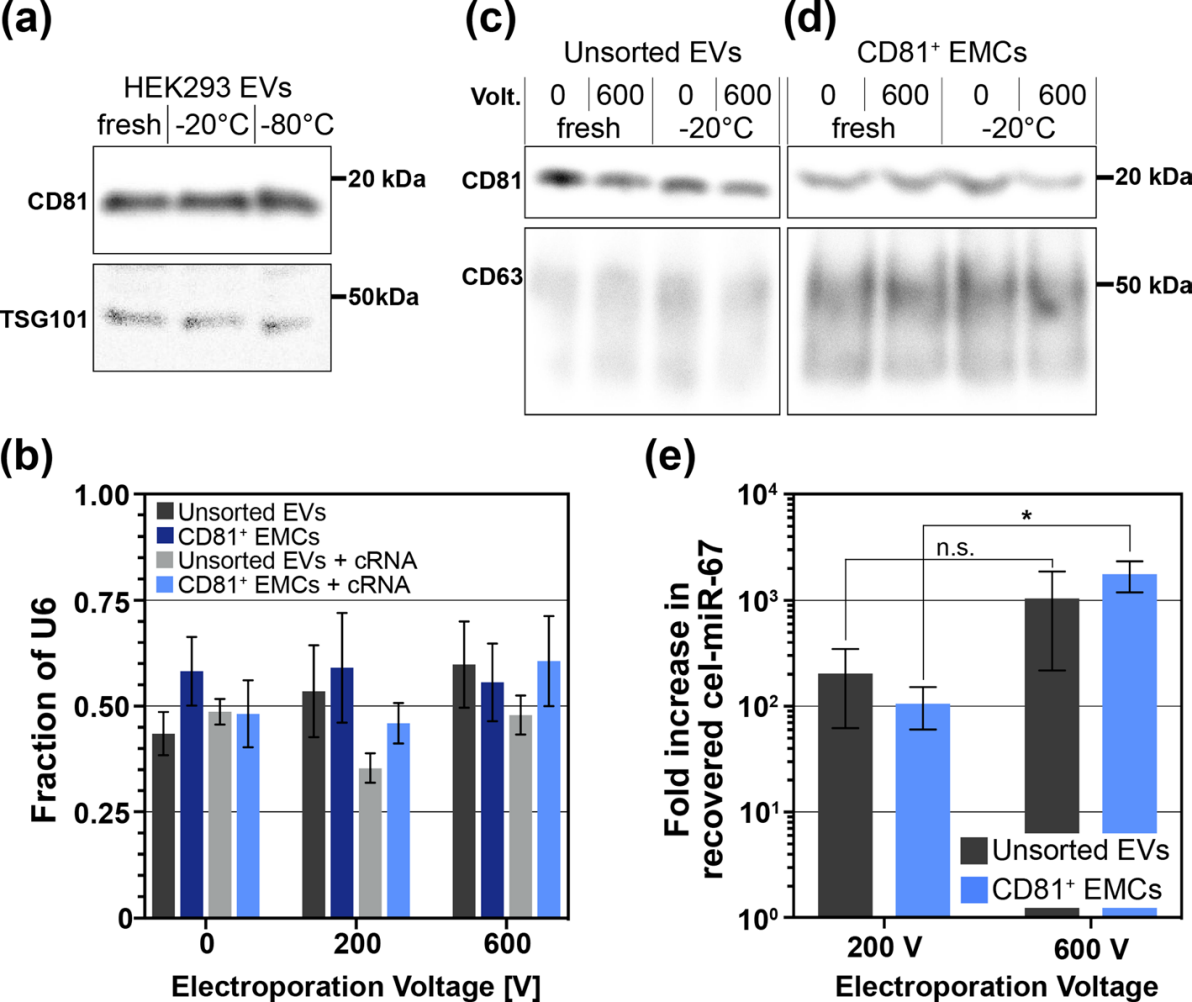
Optimized electroporation performance and the effect on EV protein and RNA content. **a** Western blot showing the effect of freezing unsorted EVs at − 20 °C and − 80 °C as compared to EVs freshly isolated from HEK-293 cell culture media. 17.5 µg of protein was loaded in each lane and TSG101 was used as a loading control. **b** EV degradation during freezing was quantified by the fraction of endogenous gene U6 remaining after freezing the EVs for 3 days at − 20 °C. Each sample of either 3 µg unsorted EVs or 1.6 × 10^5^ CD81^+^ EMCs, was split in half by volume after RNase washing for either prompt freezing or lysis followed by RNA extraction. Data is plotted as mean ± SEM. Western blot showing the effects of electroporation and freezing on **c** unsorted EVs and **d** CD81^+^ EMC protein loading. For unsorted EVs, 12 µg of protein was prepared for each sample. For CD81^+^ EMCs, 6.4 × 10^5^ EMCs were prepared for each sample. After their respective freezing and electroporation processing, EVs were lysed and 1 µg of protein, as measured with a BCA assay, was loaded into each lane. **e** Electroporation-voltage-dependent miRNA loading efficiency was determined by evaluating the change in loaded miRNA amounts via electroporation (200 V and 600 V) in comparison to the no electroporation condition (0 V) for unsorted EVs with only RNase washing and CD81^+^ EMCs with the addition of RNase, cRNA, and freezing. Relative expression of cel-miR-67 cargo miRNA was normalized to the reference gene U6 using the Pfaffl method with 0 V as the control group. Data is plotted as mean ± SEM. The Student’s *t* test (two-tailed) was performed for unsorted EVs and CD81^+^ EMCs to determine significance (*p < 0.05)

The preservation of loaded cargo and surface protein profiles of EVs throughout the entire process of EV engineering and post-modification storage is crucial to fully leverage the superior functionality of EVs as targeted therapeutic carriers. To evaluate the effects of post-electroporation frozen storage on cargo miRNA and EV surface proteins, we deliberately froze and stored electroporated EVs under harsh storage conditions of − 20 °C for 3 days in 200 µg/mL RNase buffer. After RNase incubation, CD81^+^ EMCs, with the EVs still bound to microbeads, and unsorted EVs were divided in half by volume, with half of each sample lysed for immediate RNA extraction (fresh fraction) and the other half being promptly frozen (frozen fraction). During a short-term 3-day frozen storage period, no significant changes in expression were observed in EV-selecting surface protein (CD81) and internal EV protein (TSG101) when stored at either − 20 °C or − 80 °C, as compared to EVs freshly isolated from cell culture media (Fig. 4a).

The EV membrane integrity was assessed by comparing expression levels of the endogenous gene, U6, in frozen EV fractions to those in fresh EV fractions (Fig. 4b). Comparing the fresh and frozen fractions of each sample, unsorted EVs and CD81^+^ EMCs retained on average 47 ± 2.2% and 54 ± 4.0% of U6 expression after freezing, respectively. While some loss of EVs is expected after repeated washing and freezing in these harsh conditions, we found that across all conditions, regardless of electric field strength and the presence of cRNA, there was no significant differences (*p* > 0.05) in the amount of preserved U6 after freezing (Fig. 4b).

Notably, we found that frozen storage of engineered EVs further eliminated residual unloaded miRNA, enhancing the purity of engineered EVs in Fig. 3d, e. Importantly, freezing the non-electroporated control samples (0 V) of CD81^+^ EMCs resulted in a greater reduction of unloaded residual cargo miRNA compared to freezing the 600 V samples (Figure S5b), suggesting that frozen storage primarily affects residual unloaded miRNA rather than electroporation-loaded miRNA encapsulated within EVs. For CD81^+^ EMCs, the selectively lower miRNA cargo levels for 0 V conditions, along with the preserved internal control gene expression, demonstrate that frozen storage can be conducted without substantial loss or destruction of loaded cargo in engineered EVs.

We observed that freezing the engineered unsorted EVs with cRNA resulted in comparable outcomes to electroporated unsorted EVs without the addition of cRNA (i.e., the conventional EV electroporation condition), with greatly improved significance (*p* < 0.0001, Fig. 3d). This suggests that frozen storage in RNase buffer may effectively degrade, not only residual unloaded miRNA, but also electric field-induced insoluble EV-size RNA aggregates [49, 50], which were difficult to eliminate with RNase alone. Our finding supports the hypothesis that the reduced efficiency of unsorted EV electroporation observed with cRNA addition is attributed to the carryover of excessive cRNA aggregates due to insufficient post-electroporation washing, ultimately undermining miRNA extraction yield.

Frozen storage of the CD81^+^ EMCs after electroporation did not compromise EV membrane integrity or loaded miRNA. Instead, it enhanced the purity of the final loaded-EV product from unloaded residual miRNA, allowing for accurate quantification of the true loaded miRNA amount. In addition, the combined use of cRNA and post-electroporation freezing for CD81^+^ EMCs facilitated the distinction between no electroporation control conditions (0 V) and electroporation test conditions (600 V) with a significance level of *p* < 0.0001 (Fig. 3e). Rather than directly enhancing miRNA loading, the use of RNase, cRNA, and freezing enabled the accurate detection of loaded miRNA to validate the described electroporation process for CD81^+^ EMCs by enhancing the purity of electroporated EVs from unloaded miRNA.

### Preservation of EV-selecting protein expression in miRNA-loaded EVs during frozen storage

We investigated the stability of the EV-selecting protein CD81 through immunoblotting under different EV electroporation and storage conditions. Previous work has demonstrated that electroporation of unsorted EVs in the presence of cel-miR-67 does not alter the protein composition of EVs [15]. We extended this investigation to determine if this holds true for EVs bound to microbeads during electroporation, as the impact of electroporation, presence of miRNA, and frozen storage on EVs attached to microbeads by antibody binding remain unexplored.

The use of microbeads to capture CD81^+^ EVs results in very low total protein, even after increasing EV and CD81^+^ EMCs amounts by fourfold (12 µg unsorted EVs and 6.4 × 10^5^ CD81^+^ EMCs starting material) compared to the amount used for miRNA analysis through qPCR amplification. Due to the minimal protein available for immunoblotting, only 1 µg of protein per lane was loaded, as recommended by the manufacturer’s protocol for immunoblotting EVs on microbeads (System Biosciences, EXOFLOW400A-1). Moreover, undetectable levels of cytosolic proteins (e.g., Alix [39]) led us to analyze the presence of CD81 relative to CD63, which was not used for affinity binding in our system. Immunoblotting revealed that neither electroporation alone nor the addition of exogenous RNA (cRNA and cel-mir-67) alone decreased CD81 expression in CD81^+^ EMCs (Figure S6a, b).

We also analyzed the cumulative effects of cRNA presence, electroporation, and freezing on protein expression through immunoblotting on both unsorted EVs and CD81^+^ EMCs, as shown in Fig. 4c, d. Overall, CD81^+^ EMCs retained their CD81 protein expression throughout electroporation, exogenous RNA addition, and frozen storage, as shown in Fig. 4d and Figure S6c, indicating that our EV engineering process did not damage the CD81 protein used for affinity binding. The demonstrated ability to freeze EVs after electroporation-mediated miRNA loading, even under harsh conditions, is potentially important for storage of engineered EVs for therapeutic applications. Beyond this, freezing engineered EVs after electroporation enabled loaded-miRNA amounts (Fig. 3d, e) to be accurately quantified by reducing residual miRNA without degrading encapsulated cargo miRNA, a necessary capability to effectively optimize electroporation loading parameters. The loading efficiency, integrity, and purity of EVs engineered using CD81^+^ EMCs, is coupled with the unimpeded convenience of microbead-mediated EV manipulation throughout the engineering and storage processes.

### Microbead-mediated enhanced purification enables electric field optimization

Finally, we assessed the efficiency of electroporation loading by comparing the fold change of each condition (200 V and 600 V) to the no electroporation control (0 V) for CD81^+^ EMCs under optimized conditions (RNase buffer, cRNA addition, and frozen storage) and conventional electroporation of unsorted EVs (RNase washing, without cRNA addition or frozen storage), as shown in Fig. 4e. Remarkably, with cRNA addition and freezing, electroporation efficiency for CD81^+^ EVs on microbeads proved to be comparable to the conventional method for unsorted EV electroporation (*p* > 0.05) for both 200 V and 600 V. Both unsorted EVs and CD81^+^ EMCs exhibited an increase in loaded miRNA with increasing electric field. However, only electroporated CD81^+^ EMCs exhibited a statistically significant, electric field-dependent miRNA loading efficiency between 200 and 600 V electroporation conditions (*p* < 0.05), which was not observed in unsorted EVs. This statistically-significant difference enables the ability to fine-tune the electric field to optimize cargo delivery to CD81^+^ EMCs.

Ultimately, an electric field strength of 3 kV/cm (600 V applied voltage) outperformed the loading efficiency of 1 kV/cm (200 V applied voltage) for CD81^+^ EMCs. This ability to readily adjust electroporation conditions is crucial for controlled miRNA loading dose in future studies, particularly for engineering EV subpopulations for therapeutic applications. The improved electroporation performance of CD81^+^ EMCs as compared to the unsorted EVs, paired with the significant reduction in processing time described in Fig. 3b, emphasizes the benefits of the described microbead-mediated EV electroporation loading and purification process.

## Discussion

In this study, we introduce a novel method for loading EVs with miRNA, focusing on protein-specific subpopulations using electroporation and affinity capture. Our approach integrates EV subpopulation selection, electroporation-mediated miRNA loading, and post-electroporation purification into a streamlined workflow. Key to our method is the use of affinity-based microbead manipulation, drastically reducing processing time to about 3 min per wash—a substantial improvement over conventional methods such as ultrafiltration and ultracentrifugation. This approach not only enhances cargo loading to be comparable to conventional electroporation but also allows for fine-tuning of electric field parameters for consistent outcomes. Importantly, our method minimizes capital equipment costs and eliminates the need for lengthy post-loading purification, preserving EV-selection proteins while ensuring high purity of loaded EVs with minimal residual miRNA carryover.

Our streamlined workflow enables rigorous and repetitive washings, effectively reducing the levels of unloaded miRNA to ensure the purity of engineered EVs with minimal unloaded residual cargo miRNA carryover. This rapid washing process can be tailored to minimize contamination from EVs lacking desired targeting specificity and biomolecules typically present in unsorted EV isolates [33]. By addressing challenges associated with affinity-based EV purification, our method combines purification and loading process into a single, efficient workflow. This integration eliminates the need for additional purification steps post-loading, maintaining the integrity of EV surface proteins.

Our findings highlight the limitation of repeated washing in PBS for removing residual miRNA nonspecifically bound to both unsorted EVs and CD81^+^ EMCs. While RNase treatment effectively reduced unloaded miRNA in unsorted EVs, prolonged incubation and increased washing repeats failed to adequately remove residual miRNA for CD81^+^ EMCs due to nonspecific binding on the microbeads. This persistence is attributed to strong intermolecular interactions between EMCs and RNA, as well as miRNA resistance to complete degradation by RNase [43, 44]. Despite the principle function of antibody-coated microbeads in selectively immobilizing EVs through surface targets, these antibody-coated microbeads interact extensively with various biomolecules abundant in biological solutions, leading to nonspecific interactions [51]. Affinity capture for engineering EVs thus introduces a non-specific binding challenge that cannot be fully mitigated through excessive washing and RNase incubation alone.

To overcome these challenges, we implemented a novel procedure involving the addition of carrier RNA (cRNA). The adenine-rich carrier RNA minimized nonspecific binding of cargo miRNA to exposed microbead surfaces [45]. Our approach involved a brief, 5-min incubation of CD81^+^ EMCs with cRNA, which minimally increased processing complexity for CD81^+^ EMCs compared to unsorted EVs, yet significantly enhanced the purity of engineered EVs by reducing unloaded miRNA. Interestingly, while the addition of cRNA did not improve miRNA detection in unsorted EVs due to lack of nonspecific binding to microbead surfaces, it did effectively address challenges related to affinity-based purification with the use of CD81^+^ EMCs. Through these refinements, we achieved production of highly purified, miRNA-loaded EVs with select surface proteins.

Freezing the engineered EVs for prolonged storage further enhanced the purity of the final EV product by minimizing residual unloaded miRNA, which was particularly beneficial for CD81^+^ EMCs. Previous studies have indicated that electroporation with cargo RNA does not affect the protein content of the EVs, as well as the native miRNA content [15], and EVs can protect encapsulated RNA contents for at least 30 min of incubation in RNase [52]. Our observations confirm these findings; CD81^+^ EMCs retained their characteristic protein markers and successfully protected their miRNA contents following electroporation with carrier RNA, demonstrating robust stability through subsequent RNase treatment, and frozen storage. Our refined process, involving RNase, carrier RNA, and freezing, demonstrated loading efficiencies comparable to the conventional electroporation method used for unsorted EVs while also offering enhanced capabilities to optimize electric field parameters.

While our study provides an effective method for the loading and purification of EVs, it is important to acknowledge certain limitations. Our primary focus was on simplifying the entire process of engineering EVs, encompassing EV subpopulation selection, miRNA loading, and improving the final purity of EVs, rather than exploring factors like EV concentration, miRNA levels, or the EV to miRNA ratio, which have been extensively studied [10, 15, 34, 36, 53]. We evaluated the loading under widely accepted electroporation conditions and anticipate further enhancements by optimizing critical parameters, such as the number, type, and duration of electric pulses [35, 36]. Notably, our study revealed that an electric field of 3000 V/cm (600 V) outperformed 1000 V/cm (200 V), the commonly-reported electric field strength for EV electroporation.

Further optimization possibilities exist concerning the freeze-storage conditions. Freezing at − 80 °C may mitigate damage to EVs, potentially further enhancing the longevity of intact EVs and their miRNA cargo while preserving their cellular uptake functionality [54–56]. In addition, transient EV membrane permeation during freezing could facilitate RNase penetration, reducing internal miRNA content. Although we observed no reduction in endogenous RNA expression (U6) with the addition of RNase, freezing at − 20 °C for 3 days did reduce U6 expression. This degradation could be lessened by digesting or removing RNase before EV freezing, a process simplified with the use of microbead carriers. While some EV degradation is expected as a result of the freezing process, U6 analysis may overpronounce the effects, presumably due to its longer length as compared to miRNA and known susceptibility to freeze-thaw cycles [57].

Although the rigorous removal of residual unloaded miRNA is crucial for accurately analyzing the loading efficiency and miRNA dose optimization during EV loading, such precision may not be essential for therapeutic EV delivery post-optimization. Our process prioritized the optimization of loading and purification accuracy by excluding EV elution from microbeads and lysing EVs directly on the microbeads to minimize loss and maintain precise loading detection. Given our method’s focus on handling small amounts of EVs through protein-specific subpopulation selection, even slight losses from incomplete elution could significantly affect results. For downstream therapeutic applications, efficient and gentle EV elution from the microbeads, in addition to optimized loading and purification, is critical for maintaining therapeutic quality.

Elution of immunocaptured EVs from microbeads remains a widespread challenge [58–60], although various elution strategies [59, 61–66], including some commercially-available elution buffers, such as one offered by System Biosciences (CD81 Exo-Flow Capture Kit, EXOFLOW400A-1) have been reported. While studies have demonstrated the successful delivery of functional RNA by unsorted EVs loaded through electroporation [16, 20, 22, 23, 42], confirming EV functionality and miRNA delivery efficacy after electroporation while bound to microbeads and following elution is imperative. In this study, we primarily investigated the effects of immunocapture and electroporation to validate our EV isolation and loading process, while decoupling their impacts from that of the elution process, which may further affect EV functionality, as previously observed for low pH environments used in EV elution methods [67–69]. Future work will focus on eluting the engineered EVs from the microbeads and assessing the corresponding impact on EV characteristics, including size distributions and the presence of surface and internalized proteins, to evaluate the potential of the miRNA-loaded EVs. Moreover, we will investigate the effects of distinct elution methods on miRNA-loaded EV delivery efficacy, independent of isolation and loading methods [69]. It is worth noting that the elution of EVs from microbeads may not be necessary for all applications, such as flow cytometry and microfluidic applications. Integrating our EV-loading method with microfluidic microparticle manipulation techniques is expected to significantly enhance the throughput of therapeutic EV production, advance the understanding of protein-specific EV subpopulations, and improve targeted therapeutic efficacy. Overall, our optimized process for loading miRNA into surface protein-specific EVs bound to microbeads shows promising integration potential for investigating protein subpopulations toward the development of targeted therapeutics.

## Conclusions

Our study established a fast and efficient method for engineering protein-specific subpopulations of EVs through electroporation-mediated loading of miRNA cargo. The efficacy of our workflow was demonstrated by its comparable efficiency to conventional EV electroporation methods, along with distinct advantages. Notably, our approach enables the preselection of protein-specific EV subpopulations by utilizing microbeads as carriers throughout the entire EV engineering process. This tailored strategy for loading EVs expressing specific proteins with miRNA cargo, holds promise for enhancing precision in therapeutic interventions. The innovative use of microbead carriers for manipulating EV subpopulations streamlines the workflow and reduces total post-electroporation purification processing time by about 2.5 h compared to conventional methods. This dual-selection approach offers the potential for engineering EVs tailored for the targeted delivery of gene therapies to diverse cell types while facilitating refined optimization of electroporation parameters. Furthermore, reducing processing steps improves the efficiency of EV production and minimizes contamination and variability in the final product. By refining and expanding upon this methodology, we anticipate contributing to the development of novel and effective therapeutic strategies for a range of medical conditions.

## Methods

### ***Isolation and enrichment of CD81***^+^***EVs***

HEK-293 cells were purchased from ATCC and cultured at 37 °C and 5% CO_2_ in DMEM (Gibco, 11995073) supplemented with 1% penicillin–streptomycin (Gibco, 15070063) and 10% exosome-depleted FBS (Gibco, A2720801). We employed a polymer-based precipitation kit, ExoQuick-TC (System Biosciences, EXOTC50A-1) [70], to initially isolate and concentrate the unsorted total population of extracellular vesicles (EVs) from HEK-293 cells by following the manufacturer’s instructions. HEK-293 cells were cultured in 8 mL of media in a 75 cm^2^ flask for 72 h to reach a concentration of 2 × 10^7^ cells/mL. To harvest EVs, the resulting 8 mL of cell culture conditioned media were centrifuged for 15 min at 3000×*g* at room temperature to remove cell debris. The supernatant was then combined with 2 mL ExoQuick-TC and stored upright at 4 °C overnight. The mixture was centrifuged for 30 min at 2000×*g* at room temperature to pellet EVs. After aspirating the supernatant, the pellet was centrifuged for another 5 min at 2000×*g*. The EV pellet was resuspended in 150 µL of cold, RNase-free PBS (Thermo Fisher Scientific, AM9625) and either used immediately or stored at − 20 °C for use within 2 months [53]. The presence of intact purified EVs was visualized using scanning electron microscopy, and the average diameter of the EVs was measured as 152 ± 48.5 nm (mean ± standard deviation), confirmed by analyzing 10 individual SEM images (Figure S1).

Ultracentrifugation was also used to isolate cells from HEK-293 cell culture media following the protocol described by Thery et al. [71]. Briefly, cell culture supernatant was centrifuged for 10 min at 300×*g* to remove dead cells. The supernatant was transferred to a new tube and centrifuged again for 10 min at 2000×*g* to remove any remaining dead cells and cell debris. The supernatant was again transferred to a fresh tube and centrifuged for 30 min at 10,000×*g*. Finally, the remaining supernatant containing EVs was transferred to new tubes to be centrifuged in the ultracentrifuge (Beckman Coulter Optima Max-XP) for 70 min at 100,000×*g* at 4 °C. The EV pellet was washed and resuspended in cold PBS and centrifuged a second time for 70 min at 100,000×*g* at 4 °C. This final EV pellet was resuspended in 150 µL of cold PBS.

The BCA Protein Assay (ThermoFisher Scientific, 23227) was used to quantify total protein concentrations in the EV pellets by following the manufacturer’s protocol. CD81^+^ EVs were further extracted from the isolated EVs by immunoprecipitation. An aliquot of isolated EVs containing 200 µg of total protein was conjugated with 6.4 × 10^5^ polystyrene microbeads coated with anti-CD81 antibodies (System Biosciences, CD81 Exo-Flow Capture Kit, EXOFLOW400A-1) following the manufacturer protocol. This conjugation process resulted in the formation of CD81^+^ EV–microbead complexes (EMCs). To quantify number of EMCs, the number of beads were counted using a hemocytometer, and diluted in series to analyze various concentrations of EMCs.

The EVs were stained with Exo-FITC, a proprietary reversible fluorescent dye universally staining EVs (System Biosciences, EXOFLOW400A-1), according to the manufacturer protocol. EMCs were imaged on an inverted microscope (Nikon, Eclipse Ti2) equipped with a white light source (Lumencor, Sola Light Engine) and filter cubes capable of fluorescence imaging, and a CCD camera (Photometrics, CoolSNAP DYNO). Brightfield images were taken with an exposure time of 28 ms and fluorescent images were taken with an exposure time of 1 s.

### ***Electroporation-mediated miRNA loading into unsorted and CD81***^+^***EVs***

Both unsorted EVs and CD81^+^ EMCs were subjected to electroporation using mature cel-miR-67-3p mimic (Thermo Fisher Scientific, 4464066, Assay ID: MC10867) as the cargo molecule. The EV protein to miRNA ratio, electric field strengths, and the presence of a blocking additive were considered to identify optimal loading conditions using electroporation [15, 34, 36, 53]. We prepared EVs and miRNA mixtures in a total volume of 450 µL of electroporation buffer (Gene Pulser Electroporation Buffer, Bio-Rad, 1652676) in a single microcentrifuge tube. Next, we aliquoted the mixture into electroporation cuvettes, with 50 µL for each replicate of each electroporation condition.

For unsorted EVs, each aliquot under different conditions contained approximately 3 µg of total protein and 500 ng of miRNA, following previously reported ratios [10, 15, 34, 35]. For EMCs with CD81^+^ EV subpopulation, approximately 1.6 × 10^5^ EMCs, initially incubated with 50 µg of total protein, were combined with 500 ng of miRNA in the electroporation buffer. For both unsorted EVs and CD81^+^ EMCs, a total volume of 50 µL was added to a 0.2 cm cuvette (Bio-Rad, 1652082) for electroporation at various voltages, resulting in electroporation electric fields of 0 V/cm (0 V), 1000 V/cm (200 V), 3000 V/cm (600 V), using the Eppendorf Eporator (Eppendorf, 4309000027). The electroporated unsorted EVs and CD81^+^ EMCs were promptly transferred out of their respective cuvettes into individual microcentrifuge tubes containing 5 mM EDTA to prevent nucleic acid aggregation [34]. The cuvettes were promptly washed with 50 µL TE buffer (Thermo Fisher Scientific, AM9858) to recover any remaining sample [53]. Both unsorted EVs and CD81^+^ EMCs were incubated on ice for 45 min.

### Refining EV purity through residual miRNA elimination

We employed a comprehensive three-pronged approach to eliminate unloaded residual miRNA not internalized into the EVs during electroporation loading.

1. Poly A carrier RNA blocking additive: Prior to electroporation, we introduced poly A carrier RNA (cRNA) (Qiagen, 1017647) into the electroporation buffer to block nonspecific binding on antibody-coated microbeads. The cRNA was added at a final concentration of 25 µg/mL to 200 µL of unsorted EVs or CD81^+^ EMCs in electroporation buffer and incubated for 5 min at room temperature. Next, an additional 200 µL electroporation buffer and 45 µL of cargo miRNA was added to complete the EV–miRNA 450 µL mixture prior to electroporation for a final miRNA concentration of 10 ng/µL. This strategic addition of cRNA prior to cargo miRNA addition aimed to minimize nonspecific miRNA adhesion to antibody-coated microbeads and EV surfaces during the loading process.
2. RNase wash and incubation: A meticulous RNase-based strategy involved washing and a subsequent 20-min incubation in RNase to remove and destroy unloaded cargo miRNA (either cel-miR-67 or ath-miR159a).

For CD81^+^ EMCs, the magnetic microbeads coated with electroporated EVs (EMCs) were pelleted using a magnetic stand for 3 min and resuspended in 200 µL of 200 µg/ml RNase A (Thermo Fisher Scientific, EN0531). A working solution of 200 µg/mL RNase was created by dissolving 240 µL of RNase A with a stock concentration of 10 mg/ml into 12 mL TE buffer. EMCs were incubated for 3 min in RNase at room temperature and then pelleted on the magnetic stand again.

For unsorted EVs, unloaded miRNA was removed by filtration through a 100 kDa MWCO centrifugal filter unit (Millipore Sigma, UFC210024). 125 µL of the miRNA-loaded EVs containing electroporation buffer, diluted in 1 mL of 1 × PBS, were centrifuged in the centrifugal filter unit at 7000×*g* for at least 15 min to concentrate the final unsorted EVs to a volume of approximately 20 µL. The concentrate was then resuspended and washed in a total volume of 200 µL containing 200 µg/mL RNase.

After one wash in 200 µg/mL of RNase, CD81^+^ EMCs were pelleted again on a magnetic stand, while unsorted EVs were concentrated with a centrifugal filter. Both samples were then resuspended in 200 µL of 200 µg/mL RNase and incubated at room temperature for 20 min on a rotating rack.

3. Frozen storage of loaded EVs: The electroporated EVs, both unsorted and CD81^+^ EMCs, were subjected to freezing at − 20 °C in 200 µg/mL RNase buffer for 3 days to investigate the cumulative effects of prolonged frozen storage on the degradation of unloaded miRNA and EV membrane permeability. These harsh storage conditions were chosen to maximize the degradation of unloaded miRNA and investigate the protective abilities of EVs to their miRNA cargo while bound to the microbead carriers.

The effects of the post-electroporation frozen storage were evaluated by measuring the amount of cargo miRNA internalization by electroporation loading and the gene expression levels of the EV endogenous control gene, U6 [40, 72, 73], and the protein expression of CD81, the EV-selecting protein. As a comparison to frozen samples, half of each sample by volume were prepared as fresh control samples by promptly adding 150 µL lysis buffer (Qiagen, 217204) after the 20-min RNase incubation to neutralize the RNase, readying EVs for subsequent miRNA purification.

### Quantification of electroporated miRNA via RT-qPCR

The loaded miRNA was extracted from both unsorted EVs CD81^+^ EMCs using the miRNeasy Serum/Plasma Advanced Kit (Qiagen, 217204), following the manufacturer’s protocol. After miRNA extraction, the miRNA underwent reverse transcription using Taqman MicroRNA Reverse Transcription Kit (Thermo Fisher Scientific, 4366596), followed by qPCR according to the manufacturer protocol. We selected U6, a small nuclear RNA (snRNA), as an endogenous reference gene for normalizing loaded cel-miR-67 in EV samples [40, 72, 73]. For samples without EVs, where U6 is absent (e.g., microbeads with no captured EVs), ath-miR159a served as a miRNA extraction spike-in control (Thermo Fisher Scientific, 4464066, Assay ID: MC10332) for qPCR normalization. The sequences of cargo miRNA (cel-miR-67), EV endogenous control (U6), spike-in control (ath-miR159a) are listed in Table 1. Their corresponding primers used for reverse transcription and qPCR amplifications were purchased from Thermo Fisher Scientific (4427975) and used without modification. qPCR was performed using TaqMan Universal Master Mix II (Thermo Fisher Scientific, 4440040). The primer amplification efficiencies, calculated from standard curves, were as follows: cel-miR-67, 89%, ath-miR159a, 89%, and U6, 102% (Figure S2). Due to the amplification efficiencies being dissimilar, the relative expression of the cargo miRNA to internal or spike-in controls was calculated using the Pfaffl method, an adaptation of the 2^−∆∆Ct^ method as described by Pfaffl [74]. The relative expression (RE) according to the Pfaffl method is

$${\text{RE}} = { }\frac{{E_{{{\text{target}}}}^{{\Delta {\text{C}}_{{T,{\text{target}}}} \left( {{\text{control}} - {\text{test}}} \right)}} }}{{E_{{{\text{ref}}}}^{{\Delta {\text{C}}_{{T,{\text{ref}}}} \left( {{\text{control}} - {\text{test}}} \right)}} }}$$

where *target* refers to our target gene (i.e., cel-miR-67), and *ref* refers to our endogenous control gene, U6. *E* is primer amplification efficiency for each gene. *∆C*_*T*,target_(control-target) is the difference in *C*_*T*_ value between the control group and sample test group for the target gene. Similarly, *∆C*_*T*,ref_(control-target) is the difference in *C*_*T*_ between the control group and sample test group for the reference gene.

**Table 1:** Detailed information on the RNA utilized in this study

Name	Sequence	Species	Accession number
cel-miR-67-3p	UCACAACCUCCUAGAAAGAGUAGA	Caenorhabditis elegans	MI0000038
ath-miR159a	UUUGGAUUGAAGGGAGCUCUA	Arabidopsis	MI0000189
U6 snRNA	GTGCTCGCTTCGGCAGCACATATA CTAAAATTGGAACGATACAGAGAA GATTAGCATGGCCCCTGCGCAAGG ATGACACGCAAATTCGTGAAGCGTTCCATATTTT	Human	NR_004394

To ensure data integrity, the inter-quartile range method was implemented for outlier identification. Analysis of variance (ANOVA) with Tukey’s multiple comparisons test or Student’s *t* test were performed to determine statistical significance. Statistical significance was defined as follows: no significance (n.s.); *p* > 0.05; *p ≤ 0.05; **p ≤ 0.01; ***p ≤ 0.001; and ****p ≤ 0.0001. The measured values are expressed as mean ± standard error of the mean (SEM) unless otherwise stated.

### EV membrane protein verification via western blot

The composition of the exosome protein markers tetraspanins CD81 and CD63 [39], cytosolic proteins apoptosis-linked gene 2-interacting protein X (Alix) and tumor susceptibility gene 101 (TSG101) [39, 75], and the ubiquitous cellular protein β-actin [76] in tested EV samples were assessed by western blot. Both unsorted EVs and CD81^+^ EMCs were lysed in RIPA buffer (Thermo Fisher Scientific, 89900) with 100× protease inhibitor (Thermo Fisher Scientific, 78440), followed by three rounds of sonication for 5 min each, with vortex mixing after each sonication [77]. After lysis, the protein concentration of lysates was measured using the BCA Protein Assay (Thermo Fisher Scientific, 23227). Undiluted lysates were prepared with 4× LDS sample buffer (Thermo Fisher Scientific, NP0007) and 10× reducing agent (Thermo Fisher Scientific, NP0009). Samples were heated for 10 min at 70 °C, briefly centrifuged, and then loaded into 4–12% Bis–Tris gels (Thermo Fisher Scientific, NP0321). Lanes were loaded with equivalent amount of protein or number of EMCs and their eluate, as noted in their respective figures. Gel-electrophoresis was performed with MES SDS Running Buffer (Thermo Fisher Scientific, NP0002) at a voltage of 200 V for 35 min. Proteins were transferred to a PVDF membrane (Thermo Fisher Scientific, LC2005) using transfer buffer (Thermo Fisher Scientific, NP00061) for 1 h at 30 V. Membranes underwent three 5-min washes at room temperature on a rocker with TBS-Tween20 Buffer (Thermo Fisher Scientific, 28360). The membranes were blocked for 1 h at room temperature and then incubated overnight at 4 °C on a rocker with the respective antibodies diluted in SuperBlock Blocking Buffer (Thermo Fisher Scientific, 37536). After primary antibody incubation, the PVDF membranes were again washed 3 times in TBS-T and then incubated on a rocker for 1 h at room temperature with the secondary antibody. Primary antibodies used were anti-CD63 (1:1000, Santa Cruz Biotechnology, sc-5275), anti-CD81 (1:1000, Santa Cruz Biotechnology, sc-166029), anti-Alix (1:1000, Cell Signaling Technology, 2171), anti-TSG101 (1:500, Santa Cruz Biotechnology, sc-7964), and anti-β-actin (1:1000, Santa Cruz Biotechnology, sc-47778). Secondary antibodies used were HRP conjugated sheep anti-mouse IgG (1:5000, Jackson ImmunoResearch, 515-035-003). Western blots were imaged with a GE Typhoon 5 Variable Mode Imager and were analyzed using ImageJ to perform densitometry measurements.

## Supplementary Information

Supplementary Material 1.

## Data Availability

The datasets used and/or analyzed during the current study are available from the corresponding author on reasonable request.
