# Supplementary material for: Streamlined miRNA loading of surface protein-specific extracellular vesicle subpopulations through electroporation
Source: Biomed Eng Online. 2024 Nov 21;23:116. doi: 10.1186/s12938-024-01311-2 (PMC11580418; doi:10.1186/s12938-024-01311-2)
Supplement: Supplementary file 1 — Supplementary Material 1. [file 12938_2024_1311_MOESM1_ESM.docx]

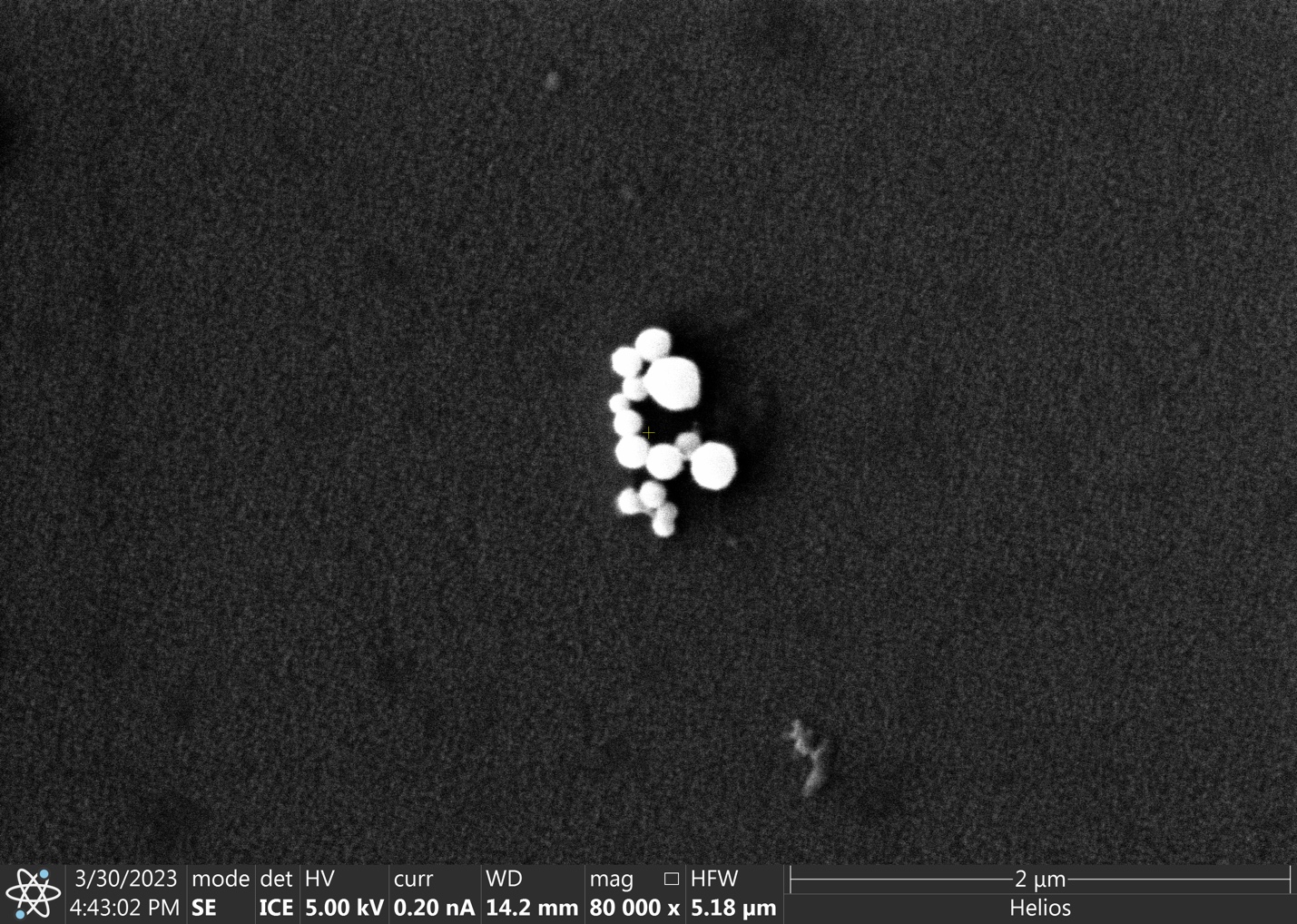


**Figure S1**: The scanning-electron microscope (SEM) image shows presence of intact extracellular vesicles. The average diameter of extracellular vesicles from 10 representative images was 152 ± 48.5 nm. Images were taken with a Thermo Scientific Helios G4 UC. To prepare the EVs for imaging, cleaned circular coverslips (Marienfeld Superior, 71861-024) were prepared with 50 µL of 1 mg/mL of poly-L-lysine solution (Sigma, P8920). After 20 minutes, the coverslips were washed two times with PBS, and then stored at 4°C overnight to completely dry. EV samples (0.14 µg/µL) were incubated overnight with coverslips in 350 µL individual wells. After incubation, the coverslips were washed four times with 1 mL of Millipore Milli-Q purified water. Next, 500 µL of 4% paraformaldehyde was added to the wells containing coverslips. After a 3-hour incubation, the wells were washed three times with 500 µL Milli-Q purified water. The samples were then sequentially immersed in 500 µL of 20, 30, 50, 70, 85, 95 and 100% ethanol solutions for 15 minutes per solution. After removing the final ethanol solution, the coverslips were dried overnight. The coverslips with fixed EV samples were sputter coated with platinum (Pt) for a total of 270 seconds. Finally, the Pt-coated EV samples on coverslips were imaged on the Thermo Scientific Helios G4 UC.

**
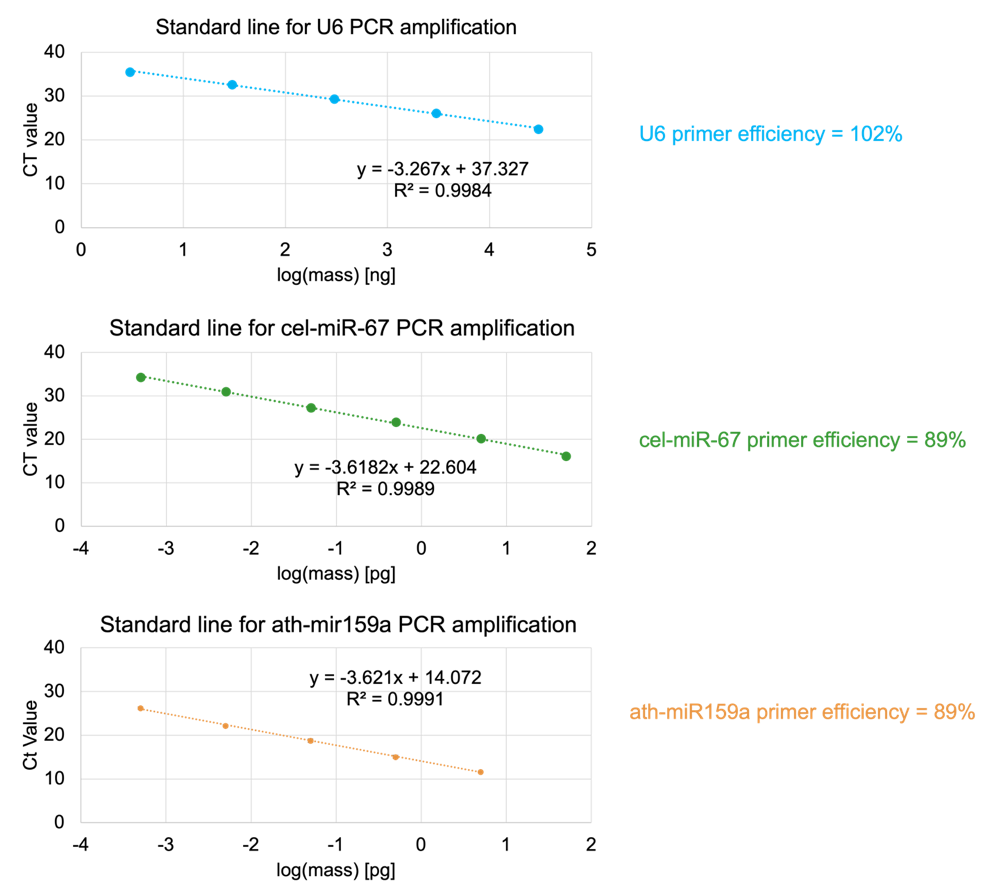
**

**Figure S2:** Primer efficiencies for U6, cel-miR-67, and ath-miR159a were calculated using standard lines created from serial dilutions of RNA. For U6, dilution of HEK-293 EV lysates were used as the control source shown on the x-axis. For cel-miR-67 and ath-mir159a, miRNA mimic purchased from Thermo Fisher Scientific were used for the serial dilutions. Efficiencies are calculated from the slope of the standard curve: Efficiency [%] = (10^(-1/slope)^-1)×100%.

**
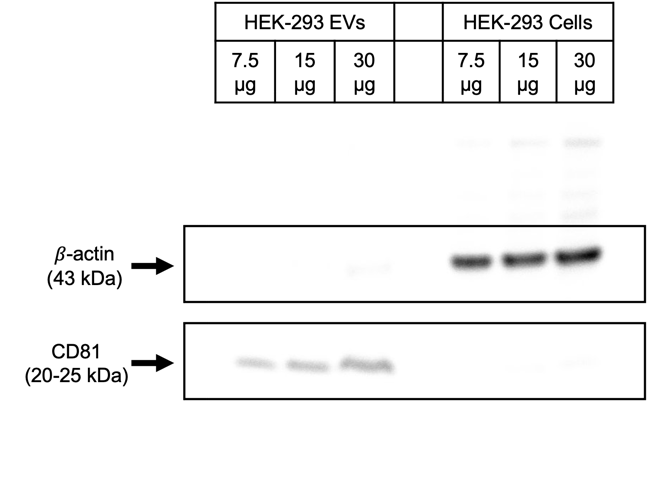
**

**Figure S3:** Full blot Figure 2b.

**Table S1:** The approximate time required for each step of the post-electroporation EV washing. EV-microbead complexes (EMCs) enable rapid EV pelleting and shorter active pipetting times than the methods required for unsorted EVs (either centrifugal filters or ultracentrifugation). Ultracentrifugation is most commonly used as an EV isolation method but requires at least 70 minutes at 100,000 x g to spin down the EV pellets.

|  | Passive/  EV pelleting | Active/  pipetting | RNase Incubation | Passive/  EV pelleting | Active/  pipetting | RNase Incubation | Total time [minutes] |
| --- | --- | --- | --- | --- | --- | --- | --- |
| EV-microbead complexes (EMCs) | 3 | 1 | 3 | 3 | 1 | 20 | 31 |
| Centrifugal filter  (unsorted EVs) | 25 | 5 | 3 | 25 | 5 | 20 | 83 |
| Ultra-centrifugation  (unsorted EVs) | 70 | 10 | 3 | 70 | 10 | 20 | 183 |


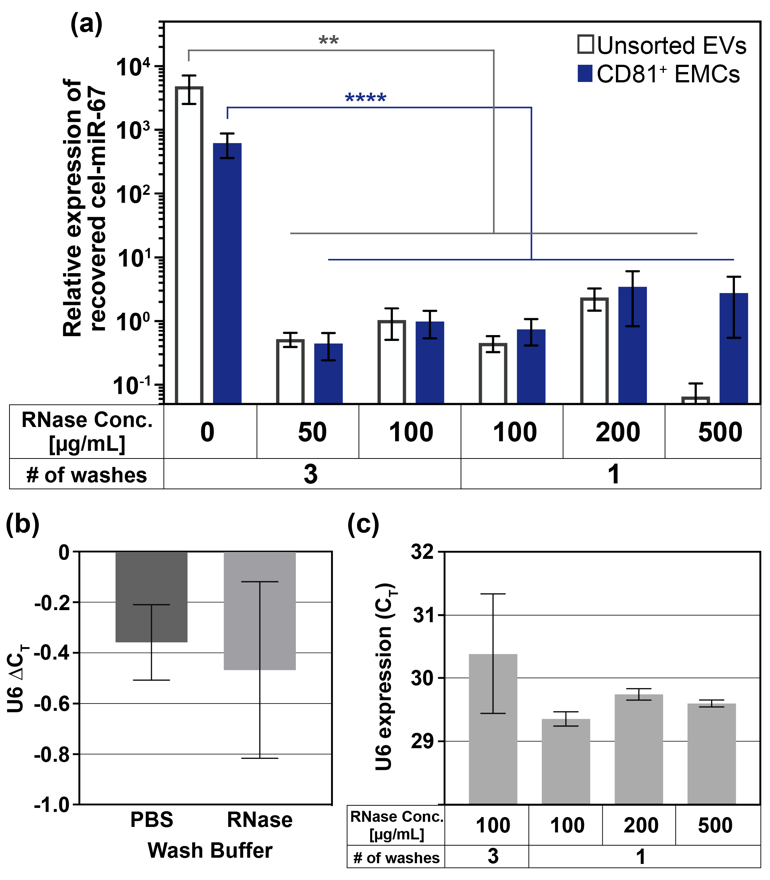
**Figure S4:** (a) The relative expression of the residual unloaded cargo miRNA (cel-mir-67) (relative to the endogenous control gene, U6) was assessed under different EV washing conditions without electroporation for both unsorted EVs and CD81^+^ EMCs. The conventional strategy of sequential washing in PBS (0 µg/mL RNase) and washing and subsequent incubation of EVs in various RNase buffer concentrations. The Pfaffl method quantified relative expression, and one-way ANOVA determined significance (**p≤0.01 and ****p≤0.0001). Values are plotted as mean ± standard error of the mean (SEM). There was no statistically significant difference between conditions with different RNase buffer concentrations or number of washes. (b) The change in U6 expression for CD81^+^ EMCs washed 3 times in PBS or RNase buffer as compared a control group of equivalent amount of CD81^+^ EMCs with no RNase incubation or washing, expressed as ∆C_T_ = C_T,control_ – C_T,wash_. A negative ∆C_T_ indicates loss of U6. CD81^+^ EMCs washed in both PBS and RNase buffer experienced a reduction in U6 expression, suggesting that this reduction is a consequence of loss of EVs during sequential washes, rather than RNase degradation of U6. (c) C_T_ values for U6 levels of CD81^+^ EMCs washed in various concentration and numbers of washes. CD81^+^ EMCs had consistent levels of U6 for increasing concentrations of RNase, but when the number of washes was increased from 1 to 3 there was a decrease in U6 expression. This supports the findings in (b) that increasing number of washes may result in loss of U6 through processing loss of EVs. Ultimately, the U6 contained in EVs does not degrade even at the high concentration of RNase up to 500 µg/mL RNase but may be lost through increasing washes.


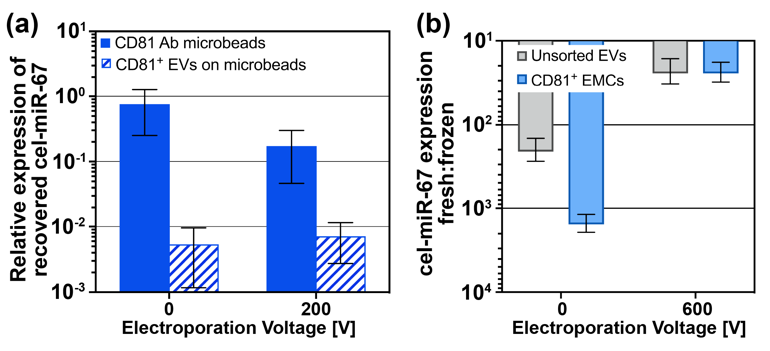


**Figure S5**: (a) The recovery of cargo miRNA cel-miR-67 was detected for both CD81^+^ EMCs and microbeads coated in anti-CD81, with no EVs bound to surface. Relative expression of cel-miR-67 is calculated by 2^-∆C^_T_ using the RNA extraction spike-in (ath-miR159a) as the control, due to lack of U6 presence for EV-absent bare microbeads. We observed that recovery of miRNA was much higher for microbeads coated only in anti-CD81 without presence of EVs, suggesting that EVs on the microbead surface prevent excessive non-specific binding of cargo miRNA to the exposed antibodies and microbead surface. Additionally, the decrease in non-specific binding with the addition for electroporation for the microbeads coated only in anti-CD81 is potentially a result of the formation of miRNA aggregates in electric field that are removed through the post-electroporation microbead washing processes. This decrease in non-specific binding is not apparent in the CD81^+^ EVs on microbeads, likely due to the successful loading of miRNA into the EVs increasing the overall recovered miRNA. The values are plotted as mean ± SEM. (b) The fold decrease between average C_T_ values for fresh and frozen samples for no electroporation (0 V) and electroporation (600 V) samples. This shows that the reduction in miRNA through freezing is much more pronounced with no electroporation (0 V), suggesting that there are high levels of unloaded miRNA present in samples, and that EVs effectively protect the loaded miRNA. Fold change is calculated using 2^-∆C^_T_ of cel-miR-67 from fresh EVs relative to frozen EVs. The mean ± SEM is shown.


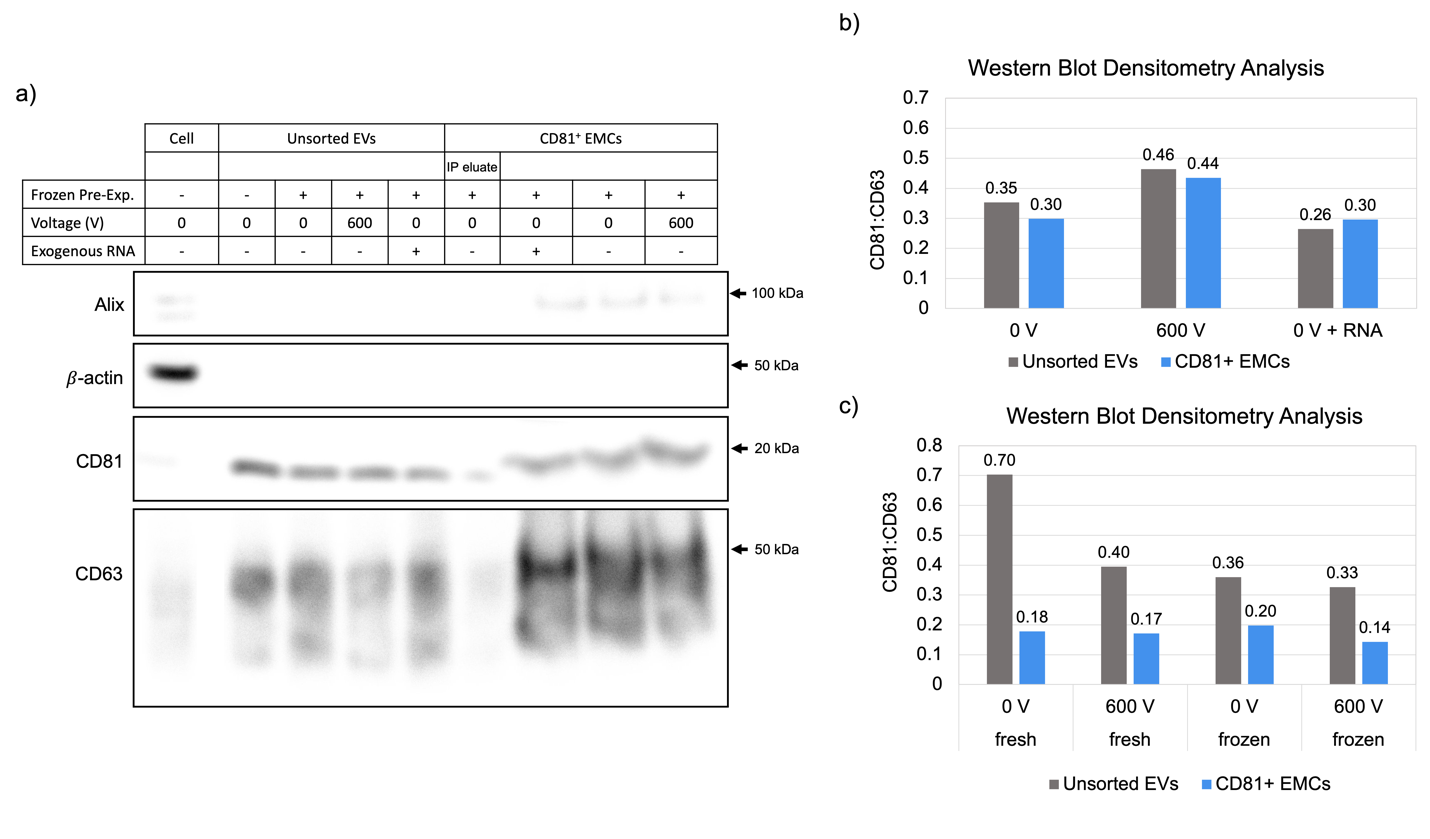


**Figure S6**: (a) Western blot shows the effect of freezing, electroporation, and the addition of miRNA and cRNA on EV protein expression. 1 µg of protein is loaded per lane due to the low total protein content of CD81^+^ EMCs. The lane “IP eluate” is the immunoprecipitation eluate (unbound EVs) after capture of CD81^+^ EVs. (b) Densitometry analysis of (a) is shown. CD81 expression is normalized to CD63 as the non-binding target exosome protein marker. (c) Densitometry analysis of Figure 4c,d. CD81 expression is normalized to CD63 as the non-binding target exosome protein marker.
